# Laminectomy for Compression Paraplegia Due to Spinal Caries

**Published:** 1892-04-16

**Authors:** 


### The Practitioner's Mirror and Retrospect.

[*The Editor will be glad to receive offers of co-operation and contributions from members of the profession. All letters should be addressed to* The Editor, The Lodge, Porchester Square, London, W.]

### SURGERY.

### LAMINECTOMY FOR COMPRESSION PARAPLEGIA DUE TO SPINAL CARIES.

The first question we have to answer in our enquiry into the value of surgical treatment in compression paraplegia from spinal caries is as to the nature of the compression. Looking at the marked angular curvature in many of these cases, we might be led to think it must be pressure from the displaced spine; but this is very rarely so. Sometimes it is from the pressure of an abscess backwards, but most commonly it is from the pressure of granulation tissue. This tubercular tissue grows usually from the posterior bodies of the vertebræ, and, perforating the posterior ligament, pushes the dura mater in front of it. It rarely penetrates the dura mater. The compression hence is exercised on the anterior part of the cord, and this is why the first symptom, as a rule, is loss of power; and if the granulation tissue grows more on one side than the other we then get paresis, at first of a unilateral character. And this is why these cases can be hopefully treated. If we had to straighten out the curvature in order to relieve the compression, any attempt would be hopeless, but if the pressure is due to granulation tissue we can remove that, or if it is an abscess pressing on the cord we have a better chance still of doing good, for it is easier to evacuate an abscess than to be sure of getting away all the tubercular tissue. The cord may be compressed after a time by this tubercular granulation tissue when it has undergone further change into fibroid thickening, and this is the kind of tissue Macewen, of Glasgow, has had to remove in some of the cases he has operated on with such remarkably good results. Sometimes, fortunately, he has found this tissue between the dura mater and the laminæ, a position in which it is much easier to remove it than when present between the bodies of the vertebræ and the cord. Of course it would be much easier to get at the granulation tissue on the anterior aspect of the cord in some regions of the spine than in others. In the dorsal region we have found in adults there is about one inch between the nerve roots, and it would not be a serious matter to divide one root here to get more room, as Horsley did in removing his meningeal tumour. It would be more difficult to get at the anterior aspect of the cord in the region of the cervical enlargement, where so many nerve roots come off. In the region of the lumbar enlargement, although so many roots come off, they pass so nearly directly downwards by the side of the cord that they would not seriously block the way. They pass out through the intervertebral foramina lower down, and do not cross the canal at the level of the enlargement.

Lane, at the Clinical Society, in October last, brought forward eleven cases of laminectomy for spinal compression, and in every case but one the pressure was due to an abscess connected with the spinal caries. This was an unusually fortunate condition to treat. The fibrous tissue growth at the back of the cord which Macewen found, was not present in any of these cases. He says, "Several of these cases would of a certainty have died from chest or bladder complications, which only disappeared when they recovered power over their intercostal and abdominal muscles." He did not find the tubercular tissue grow so rapidly after removal as to quickly compress the cord again. There is, however, one advantage which he claims for laminectomy which seems rather surprising. He says, "It enables the surgeon to treat the diseased vertebræ directly, not only by spooning, irrigation, and the thorough removal of all carious material and diseased bone, but also by the repeated application of iodoform." Now, as Bowlby pointed out at the meeting, it must be a very difficult matter, indeed, for we have to work around the cord and nerve roots. It must be difficult enough to get away the tubercular granulation tissue, much more carious bone. Bowlby's experience was that it was very difficult to take away more than the merest fragments of carious bone. However valuable the operation may be to relieve compression of the cord, we can, surely, hardly hope to get away diseased bone through the spinal canal. But, possibly, Lane means that besides the operation of laminectomy he was able at the same time to get away carious bone round the sides of the vertebræ as in Treves' lumbar opening for psoas abscess, through which we have seen quite large sequestra removed.

The question when we ought to operate in cases of compression paraplegia is a very important one. Looking at the good results which he obtained, Lane advises operation with as little delay as possible. But the experience of most surgeons is that some cases of the worst kind, cases where compression has involved the whole thickness of the cord, and of long duration, will recover without any operative interference. Watson Cheyne has seen several cases recover after prolonged rest, which were taken into hospital for laminectomy. If, however, the bladder paralysis is accompanied by cystitis, with the danger of surgical kidney, or the breathing is seriously interfered with by paralysis of the intercostal nerves, then we are not justified in waiting and hoping for gradual absorption or sclerosis of the tubercular granulation tissue. Or if the disease has gone on for years, all active mischief has subsided, and yet the patient is left paralysed, and with no control over the bladder, as in some of Macewen's cases. Here, again, we are justified in performing laminectomy, and we shall very likely find the compression due to the fibrous tissue formed from old tubercular granulations. A hectic temperature, indicating the possibility of a generalised tuberculosis contra-indicates laminectomy. Macewen will not operate under such conditions.

Chipault has lately operated on thirty-five cases of compression paraplegia due to spinal caries, and in twenty there has been a cure or marked improvement. In those cases which had not been benefited by operation it seemed as if the laminæ had not been sufficiently removed to allow of complete scraping out of the tubercular granulation tissue; in some, perhaps, the degeneration changes in the cord were too advanced to permit of recovery after removal of the pressure, and yet Macewen's cases of recovery were of a very long duration. As usual in transverse lesions of the cord, an ascending degeneration in the sensory tracts and a descending degeneration in the motor tracts, especially the crossed pyramidal tract, follow the softening which involves the whole thickness of the cord at the seat of pressure when far advanced.

Only three or four of Chipault's cases died directly from the operation; in these the pressure was high up in the cord, and it was injured in the operation. In the cases that recovered improvement began in a few days, sometimes in a few hours. Sensation returned before motion, and the sphinctus quickly regained power.

The symptoms of slow compression of the cord from spinal caries are well given in the last edition of "Erichsen's Surgery." The diagnosis is not difficult when we have the excurvation to guide us. This is not, however, invariably present, and then we may be unable to decide on the nature of the compression so easily, though the pain in the spine on stooping, and pressure downwards, and rocking of the shoulders, and the way in which symptoms begin—pain, then motor weakness, and then sensory failure, will point to compression of the cord from Potts' disease, the pressure acting first on the nerve roots and anterior tracts. As we described in a recent paper, meningeal tumours often cause a feeling of weakness in the back, and even tenderness.

The method of performing laminectomy we described in a paper on tumours of the membranes of the spinal cord. In operating for pressure from spinal caries, however, we always find the lesion extra-dural, and hence have no need to open the dura mater. For cutting away the tubercular tissue on the front of the cord, we would suggest the use of small scissors, like iridectomy scissors, much curved on the flat.

